# Affective reserve: A new reserve concept we should talk about

**DOI:** 10.1371/journal.pmen.0000083

**Published:** 2024-07-12

**Authors:** Elisa Di Rosa

**Affiliations:** Department of General Psychology, University of Padova, Padova, Italy; PLOS: Public Library of Science, UNITED KINGDOM OF GREAT BRITAIN AND NORTHERN IRELAND

When talking about “reserve” in psychology, the concept of Cognitive Reserve inevitably comes into mind. Specifically, Cognitive Reserve (CR) has been defined as “the adaptability (i.e., efficiency, capacity, flexibility) of cognitive processes that helps to explain differential susceptibility of cognitive abilities or day-to-day function to brain ageing, pathology, or insult” [1]. CR is often referred to as the “software”, while the “hardware” would be represented by the concept of ‘Brain reserve’, i.e., the structural characteristics of the brain such as number of neurons and synapses for which individual variations “allow some people to better cope with brain ageing and pathology than others before clinical or cognitive changes emerge” [1]. The main application of the CR theory has been the study of cognitive changes due to neurological diseases, largely neurodegenerative processes resulting in dementia, as well as to healthy ageing, with evidence identifying a positive association between CR and cognitive performance.

The present opinion has been inspired by the evidence investigating the interaction between cognitive and affective states and dispositions (i.e., emotion, mood and motivation) [2–4] as well as by the numerous studies showing that affective disorders, such as depression, anxiety and stress related disorders, can represent a risk factor for the development of cognitive decline and dementia [5–7]. This set of evidence stimulated several reflections about the need to go beyond the CR construct to better understand the variability in both cognition and affect. Hence, this Opinion will propose a new reserve construct, i.e. the Affective Reserve.

## Affective reserve: Proposing a new definition

Affective Reserve can be defined as the *capacity to regulate affective states and dispositions (i*.*e*., *emotions*, *mood*, *motivation) that helps to explain the differential susceptibility and response to stressful and adverse life events*, *pathologies*, *and ageing*.

Importantly, this is a new definition of Affective Reserve, which in the literature was previously proposed only twice and in the context of ageing. Precisely, in 1998 Gatz [8] suggested a model of late-life depression whereby both genetic and environmental factors contributed to physical and depressive symptoms in older adults, and where fatigue corresponded to low Affective Reserve. Twenty-five years later, Mohindru and colleagues [9] suggested that Affective Reserve would be “the ability (of older adults) to adapt to stressful situations and prevent the impact of emotional distress… as a plausible outcome of the age-related positivity effect”, i.e., the tendency of older adults to focus more attention on positive information, rather than negative [10].

The construct of Affective Reserve, however, should not refer to ageing only, as it might be applicable to other potential causes of changes in affect and cognition, such as pathologies, as well as stressful and adverse life events.

Hence, together with a new definition, a new model of Affective Reserve is here proposed, assuming that the same adverse life event, pathology, or ageing process can bring a different response in each person, and that each response will be also determined by the individual variability in the capacity to regulate emotions, mood, and motivation, i.e., in their Affective Reserve level.

In this new conceptualization, affect-regulation strategies such as emotional and motivation regulation, coping behaviours, but also social support, might represent proxies of Affective Reserve.

## Beyond emotional and motivational reserves

The proposed model of Affective Reserve has been developed with the aim to be more comprehensive and, moreover, go beyond the two existing constructs of Emotional and Motivational Reserve.

Emotional Reserve has been defined as “the adaptability of the individual emotional functioning, which helps to explain individual differences in affective functioning, as well as in the processing of emotional information when facing emotionally challenging situations” [11].

Motivational Reserve has been defined as “a set of motivational abilities that provide individuals with resilience to neuropathological damage” [12, 13].

The new construct of Affective Reserve, indeed, is not only defined as the capacity to regulate emotion, motivation, as well as mood, but is also providing a comprehensive framework, where those different affective states and dispositions are not considered independently, but as part of the same reserve mechanism, as they are all embedded in the individual response to stressful and adverse life events, pathologies, and ageing.

As a comparison, the CR construct refers to the adaptability of all the different cognitive processes, without considering, for example, attention and memory or language in a separate way. The same principle can be applied to the concept of Affective Reserve, which refers to the capacity to regulate affective states and dispositions, without considering emotion, mood and motivation in a separate way.

Affective Reserve is therefore not just a new label to cover two existing concepts, but a different construct that goes beyond them, providing a comprehensive and unitarian framework that could also result in more efficient assessment approach for both researchers and clinicians.

## Affective reserve as predictor of resilience

Finally, Affective Reserve, as described in this new model, can be considered a possible predictor of Resilience, a term that has been introduced in the psychological literature to explain the interindividual variability in response to stressful and adverse life events, pathologies and ageing.

Within the multiple definitions and frameworks of Resilience, two of the most recent ones show indeed a clear link with the new Affective Reserve construct.

The first is the Affect-regulation framework [14], where coping and emotion-regulation strategies are considered a key factor for Resilience, defined as a “better-than-expected psychological health”, i.e., a health outcome that can correspond to lower levels of psychopathology, fewer symptoms or absence of diagnosis after adversity exposure [14].

The second is the one suggested in the context of CR [15], where Resilience has been defined as “a general term that subsumes any concept that relates to the capacity of the brain to maintain cognition and function with aging and disease”, related to variables underlying mechanisms [1] and to multiple reserve processes [1].

Hence, despite the differences between these two accounts, the clear link between their definition of Resilience and the concept of Affective Reserve highlights the utility of this new reserve construct in the study of affective and cognitive processes, in both healthy and pathological conditions.

## To summarise, affective reserve is

- a new reserve construct to that helps explaining the differential susceptibility and response to stressful and adverse life events, pathologies, and ageing
- a reserve mechanism applicable in the study of both affective and cognitive disorders
- a unitarian construct that goes beyond the concepts of Emotional and Motivational Reserve
- a possible predictor of Resilience

## Conclusion and future directions

The idea presented here is offered as a new conceptual framework for defining and studying Affective Reserve. Several pathways can be covered by mental health research to empirically corroborate this novel construct, and to allow its possible evolution. For example, understanding the relationship between Affective and Brain Reserve would be extremely important to understand its neurobiological foundation. Also, evaluating possible links between Affective and Cognitive Reserve might reveal interesting phenomena and contribute to the study of the interaction between affective and cognitive processes. These are just some questions that this Opinion aims to stimulate, with answers that will hopefully bring important steps forward in mental health research and clinical practice.
